# Confined Dynamics of Water in Transmembrane Pore of TRPV1 Ion Channel

**DOI:** 10.3390/ijms20174285

**Published:** 2019-09-01

**Authors:** Yury A. Trofimov, Nikolay A. Krylov, Roman G. Efremov

**Affiliations:** 1 M.M. Shemyakin & Yu.A. Ovchinnikov Institute of Bioorganic Chemistry, Russian Academy of Sciences, Miklukho-Maklaya Street, 16/10, 117997 Moscow, Russia; 2 National Research University Higher School of Economics, Myasnitskaya ul. 20, 101000 Moscow, Russia; 3 National Research Nuclear University Moscow Engineering Physics Institute, Kashirskoe Shosse, 31, 115409 Moscow, Russia; 4 Moscow Institute of Physics and Technology (State University), Dolgoprudny, 141701 Moscow, Russia

**Keywords:** anomalous water diffusion, computer simulations, ion channel gating, molecular dynamics, nano-size water pore, physico-chemical properties of confined water, protein-water interactions, TRPV1 channel permeability for water, water dynamics, water H-bonding

## Abstract

Solvation effects play a key role in chemical and biological processes. The microscopic properties of water near molecular surfaces are radically different from those in the bulk. Furthermore, the behavior of water in confined volumes of a nanometer scale, including transmembrane pores of ion channels, is especially nontrivial. Knowledge at the molecular level of structural and dynamic parameters of water in such systems is necessary to understand the mechanisms of ion channels functioning. In this work, the results of molecular dynamics (MD) simulations of water in the pore and selectivity filter domains of TRPV1 (Transient Receptor Potential Vanilloid type 1) membrane channel are considered. These domains represent nanoscale volumes with strongly amphiphilic walls, where physical behavior of water radically differs from that of free hydration (e.g., at protein interfaces) or in the bulk. Inside the pore and filter domains, water reveals a very heterogeneous spatial distribution and unusual dynamics: It forms compact areas localized near polar groups of particular residues. Residence time of water molecules in such areas is at least 1.5 to 3 times larger than that observed for similar groups at the protein surface. Presumably, these water “blobs” play an important role in the functional activity of TRPV1. In particular, they take part in hydration of the hydrophobic TRPV1 pore by localizing up to six waters near the so-called “lower gate” of the channel and reducing by this way the free energy barrier for ion and water transport. Although the channel is formed by four identical protein subunits, which are symmetrically packed in the initial experimental 3D structure, in the course of MD simulations, hydration of the same amino acid residues of individual subunits may differ significantly. This greatly affects the microscopic picture of the distribution of water in the channel and, potentially, the mechanism of its functioning. Therefore, reconstruction of the full picture of TRPV1 channel solvation requires thorough atomistic simulations and analysis. It is important that the naturally occurring porous volumes, like ion-conducting protein domains, reveal much more sophisticated and fine-tuned regulation of solvation than, e.g., artificially designed carbon nanotubes.

## 1. Introduction

The behavior of water near molecular surfaces is known to be critically important in chemistry and biology [1,2]. Thus, in a protein hydration shell, it presumably plays a sufficient role in many biophysical processes and defines pivotal protein properties, like protein folding (water expulsion from a hydrophobic core mediates the rate of the late stage folding [1]); ligand binding (water molecules can affect the selectivity of a binding site [2]); protein aggregation (accelerated water dynamics speeds up the growth of aggregates by facilitating the binding of new peptide monomers [3]); thermal stability (strong protein–water interactions prevent unfolding of thermophilic proteins [4]), and so on. Nontrivial dynamics of water was also reported on the water/membrane interface and inside lipid bilayers [5]. These effects were associated with the functioning of cell membranes, for example, with their permeation for water and other substances, adsorption of proteins, protein–protein interactions in membranes, etc.

Experimental studies and atomistic simulations showed that water molecules in a solute hydration shell (e.g., proteins, membranes) have retarded dynamics compared to the bulk water by a factor ≈2 to 6 [6]. Such a slowdown can be explained by the extended molecular jump mechanism. It describes dynamics of water molecules as fast large-amplitude rotational jumps occurring due to the exchange of hydrogen bonded (hb) partners, and a relatively slow diffusion process occurring between the jumps [7]. The typical time constant of molecular rotational motions and hb lifetimes is about 2 ps [8] for bulk water. In the jump model, two factors affect water dynamics in solute hydration shell. The first one is the excluded volume effect that is caused by reducing the number of probable hb partners for water molecules due to the water–solute interface topology. The typical slowdown factor of water in this case is less than two at the local convex solute sites and exceeds two at the concave sites [9]. The second factor is water–solute hb interactions. This can change the rotational dynamics of water depending on the type (hb donor or acceptor) and strength of these bonds in comparison with the water–water hb [10]. The largest deceleration effect is induced by strong hb acceptors, such as carboxylates, whose slowdown factor also exceeds two.

Both factors have a strong influence on the dynamics of water confined in artificial and natural nanoscale channels, where the aforementioned “water braking” is much more pronounced. Thus, at the wall of a relatively broad (ø 24 Å) hydrophilic silica channel, water rotational dynamics are lengthened by roughly two orders of magnitude compared with the bulk phase [11]. These dynamics assume a broad distribution of rate constants that arises from the spatial heterogeneity of the channel surface [12]. While in the internal core region of the channel, nearly bulk-like homogenous dynamics are observed.

In narrow channels (e.g., carbon nanotubes), water molecules are often arranged in a single file and move as a unit due to strong hb interactions with each other. Water rotational dynamics in such channels are slowed down to the order of several nanoseconds [13]. For porous microstructures, like perturbed channels [14] and water permeable membranes [15], the interaction between the channel wall and water strongly effects the water hb–network (and water dynamics) due to the confinement and affinity of channels or membrane polar groups. Water permeability through such structures critically depends on their molecular design. For example, water transfer resistance of aromatic polyamide membranes can be reduced by enhancing the interfacial hydrophilicity and the interior hydrophobicity of their structures [15].

Besides the above said artificial chemically synthetized nano objects, it is very important to understand the detailed water behavior in the naturally occurring ones—e.g., in transmembrane (TM) protein ion channels. For the latter, spatial arrangement of hydrophobic/hydrophilic properties of the pores is often critical for their functionality. Thus, hydrophilic residues in the pore of aquaporins (a family of proteins, which provide rapid and highly selective conduction of water and other small molecules across the cellular membrane) lower the energy barrier for water permeation by offering replacement interactions to water molecules in order to compensate the loss of water–water hydrogen bonds [16]. An opposite example of this hydrophobic influence on the channel’s activity is the so-called hydrophobic gating. In this case, a hydrophobic constriction in a channel pore mediates an energetic barrier to water and ion permeation by means of partial hydration/dehydration of the pore [17,18].

In this work, the effects of the biological pore on water dynamics were studied using the results of molecular dynamics (MD) simulations of water in confined conditions of the pore and filter domains of the TRPV1 ion channel. Vanilloid receptor TRPV1 (Transient Receptor Potential Vanilloid type 1) is a non-selective cation channel that accomplishes transport of ions and water molecules through a cellular membrane in response to capsaicin, temperatures above 42 °C, and other physico-chemical stimuli [19]. TRPV1 is preferentially expressed in neurons of the peripheral neuron system responsible for sensation to heat, hot spice, pain, itch, and so on [19,20].

TRPV1 is composed of four identical subunits, which form a pathway for ions and water molecules in a lipid bilayer [21,22,23]. This pathway has two “bottlenecks”: The so-called upper gate that is formed by a selectivity filter created by short unstructured segments at the extracellular side of the channel and the lower gate located in the bundle composed of four crossing TM helices S6 disposed closer to the cytoplasmic side. The narrowest place of the upper gate is formed by carbonyl oxygen atoms of the residue Glu643 (the distance between the atoms of diagonally located subunits is 7.6 Å in the open state [22]). The lower gate bottleneck is formed by side chains of Ile679 (7.6 Å in the open state [22]).

The cavity between the gates (the pore) has mostly hydrophobic walls [24]. However, few polar groups here are exposed to solvent: Backbone carbonyls of some residues, hydroxyl group of Thr671, and polar groups of Asn676. Such an organization of the TRPV1 pore represents a rather unusual natural nano object—strongly amphiphilic confined volume with presumably flexible walls formed by four TRPV1 subunits stabilized by the membrane environment. It is reasonable to assume that the dynamic behavior of water may drastically differ from that in bulk water as well as on the protein or membrane interfaces.

## 2. Results

**Figure 1:**
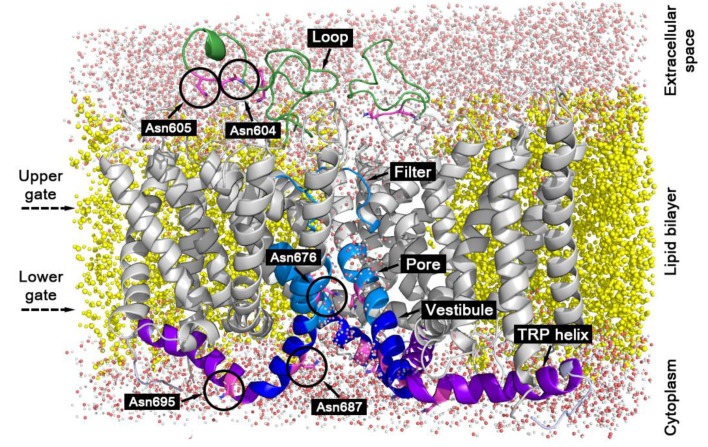
Ribbon representation of TM segments of TRPV1 embedded into hydrated lipid bilayer (three protein subunits are shown). The protein domains considered in the analysis of water dynamics are colored: green—extracellular loops (residues 604–625), blue—pore segment of the helices S6 (residues 670–680), and selectivity filter (residues 642–646), dark blue—vestibule segment of the helices S6 (residues 681–692), purple—TRP helices (residues 693–711), gray—other protein parts. Asparagine residues of TRPV1 further mentioned in the text are marked by black circles. Yellow—lipid bilayer, red and white dots—water molecules. Dashed arrows mark the levels of the upper and the lower gates.

To investigate the dynamics of confined water in TRPV1, the most spatially limited protein domains were chosen: The pore segments of helices S6 between the upper and lower gates and the selectivity filter (residues 670–680 and 642–646, respectively, Figure 1). For comparison, three additional domains were considered: Pore vestibule, the less confined volume, which is formed by the segments of helices S6 under the lower gate (residues 681–692); TRP helix lying on the cytoplasmic side of the membrane and strongly exposed to the bulk water (residues 693–711); loops in the extracellular entrance of the channel—they represent protein regions, which are mostly exposed to bulk water in TRPV1 (residues 604–625).

### 2.1. Translational Dynamics of Water along the Pore Axis

**Figure 2:**
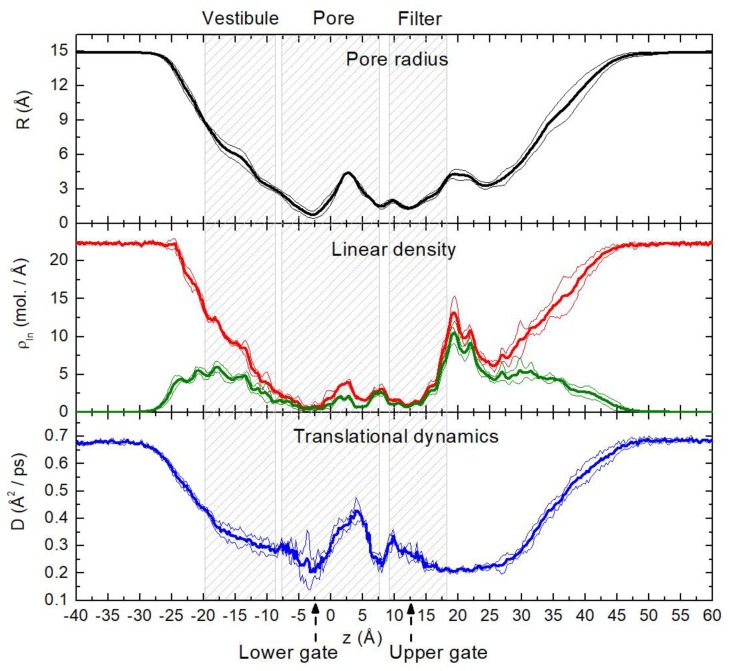
Pore radius (*R*), linear density (*ρ_ln_*), and translational dynamics coefficient of water (*D*) along the pore axis (*z*). Linear density of all water molecules and waters bound to protein polar groups via hb are shown with red and green, respectively. Thick and thin curves display average values (calculated over four MD trajectories) and standard deviations, respectively. Hatched areas indicate vestibule, pore, and filter domains of the protein. TRP helix and loop domains are out of the boundaries of the volume under consideration (cylinder with the radius of 15 Å). Dashed arrows mark the location of the upper and the lower gates.

Figure 2 shows the pore radius (R), water linear density (ρ_ln_) for all water molecules (red curve) and for molecules bound to protein polar groups via hb (green curve), and translational dynamics coefficient (D, see Materials and Methods). All these profiles were calculated in a cylinder (ø30 × 100 Å) oriented along the pore axis. The profiles were averaged over four MD trajectories. Minima of the curves, R(z) and ρ_ln_(z), in the regions of *z* = −5…0 Å and 10…15 Å correspond to the narrowest zones of the pore: The lower and upper gates. It can be seen that water in the confined protein domains has about 1.5 to 3 times slower translational dynamics in comparison with the bulk (*z* < –30 Å and *z* > 50 Å). Above and below the gates, the mobility of water molecules increases as the channel expands. The maximum of D in the pore domain (about 0.4 Å^2^/ps at *z* = 3…5 Å) corresponds to the maximum of the pore radius (*R* = 3.5…4.5 Å). In this region, there is a large cavity located between the gates, where about a half of the water molecules do not form hb with the protein. Furthermore, the maximum of D(z) is probably related to the formation of a bulk-like phase similar to the core water in silica nanotube in [11]. An opposite scenario can be seen in the range of *z* = 15…30 Å. Very slow water dynamics (D ≈ 0.2 Å^2^/ps) take place in spite of a relatively large pore radius and water density. Such a slowdown is caused by the unstructured protein regions, which evenly fill about half of the volume in this region and intense water–protein hb interactions (about 75% of water molecules are bound to protein).

### 2.2. Spatial Distribution of Water in the Pore

**Figure 3:**
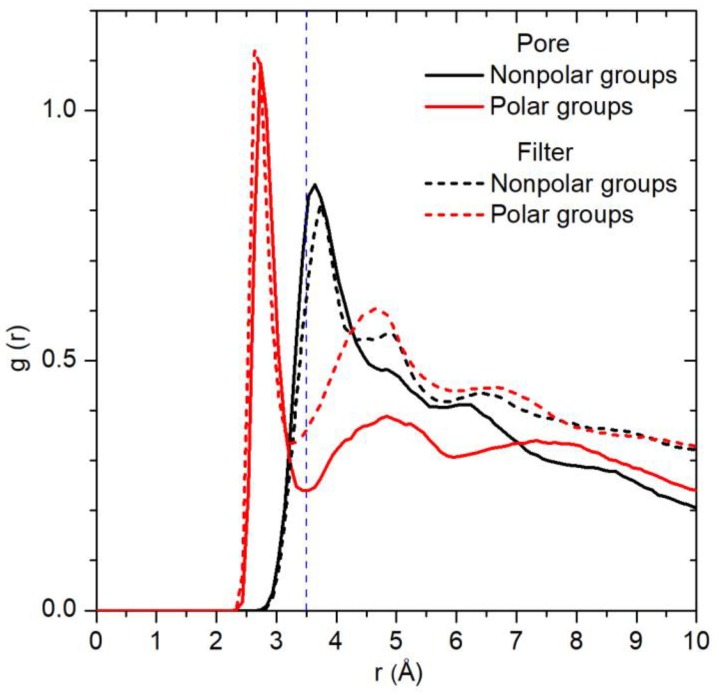
Radial distribution functions of water oxygens around polar and nonpolar groups of the pore and filter domains. The most hydrated groups of Gly643, Met644, Gly645, Asp646 (filter) and Tyr671, Asn676, Ile679, Ala680 (pore) were taken for calculation. *r* = 0 corresponds to the position of the central atom of a group (oxygen or nitrogen for polar groups and carbon for nonpolar groups). Dashed vertical line shows the radius of 3.5 Å around the protein groups.

Water distribution around particular protein groups (atoms) can be investigated with the radial distribution function (g(r)) [25]. This function for water oxygen atoms calculated around polar (and charged) and nonpolar groups of the pore and filter domains is shown in Figure 3. For analysis, the groups with coordination number ≥ 0.2 were chosen. It can be seen that around polar groups there is a pronounced peak within the radius of 3.5 Å. Because nonpolar groups reveal a broader peak shifted by 1 Å from the center, they form a low density region in their 3.5-Å neighborhood. The difference between the water radial distribution around polar and nonpolar groups can be explained by the water–protein hb interactions, which preferably retain water molecules near the polar groups.

**Figure 4:**
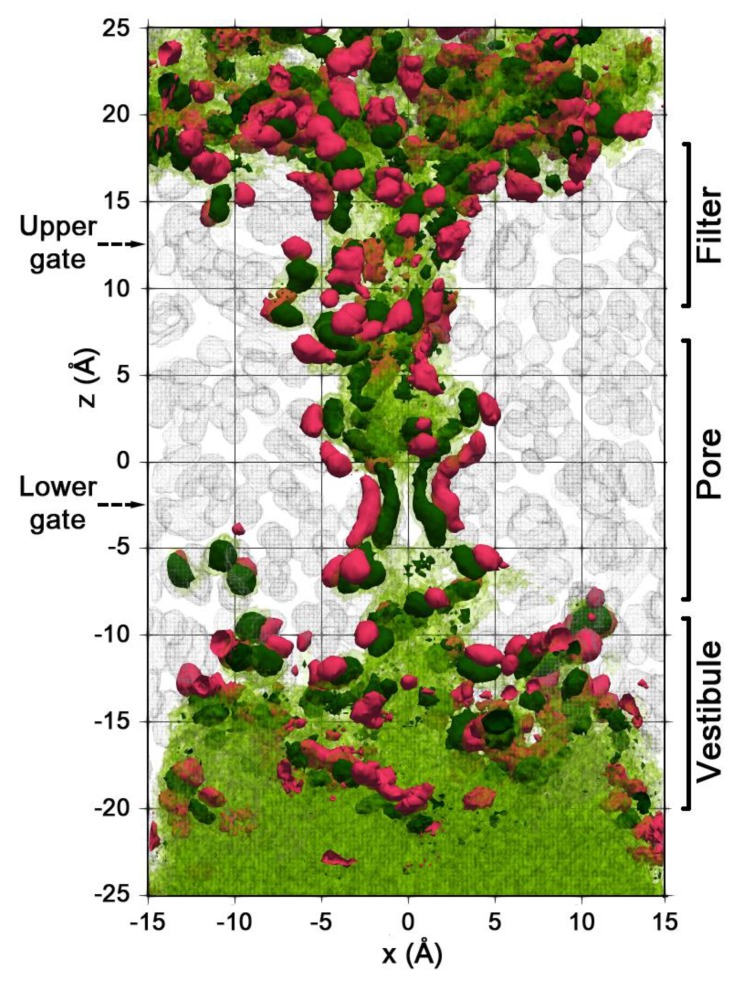
Spatial density distribution of protein atoms, water molecules, and protein–water hb in the cylinder (ø30 × 50 Å) along the pore axis averaged over one of the trajectories. Gray—isosurface of protein density *ρ* = 0.1 atom/Å^3^ (part of the protein is removed for clarity), dark green—water with ρ = 0.1 mol/Å^3^, green semi-transparent—water with density similar to the bulk water *ρ* = 0.03 mol/Å^3^, pink—water-protein hb with *ρ* = 0.1 bound/Å^3^. Boundaries of the vestibule, pore, and filter domains are shown on the right. Dashed arrows mark the levels of the upper and the lower gates.

This water molecules’ localization can be seen in Figure 4, which shows the averaged spatial density distribution of water, protein, and water–protein hb. Isosurfaces corresponding to densities of (ρ) 0.03 (semitransparent green) and 0.1 mol/Å3 (opaque dark green) are indicated. The former corresponds roughly to the bulk water density. The second shows the areas of anomalously high water density (water localization areas). High-density areas are preferably disposed near the protein polar groups, which manifest themselves via water–protein hb interactions (pink areas in Figure 4).

A very heterogeneous water spatial distribution with a high abundance of water localization areas can be seen in the pore and filter domains, while in the less confined vestibule there is a more homogeneous water distribution. This picture corresponds to the linear density of water along the pore axis in Figure 2, where about 70% of the 60 water molecules in the pore and filter domains are bound to protein polar groups, while in the vestibule domain, only 50% of the 96 waters are bound. Moreover, the contribution of bonded molecules decreases to 36% in the bottom part of the vestibule (*z* = –20 Å).

### 2.3. Water Residence Time in the Hydration Shells of Polar and Nonpolar Protein Groups

**Figure 5:**
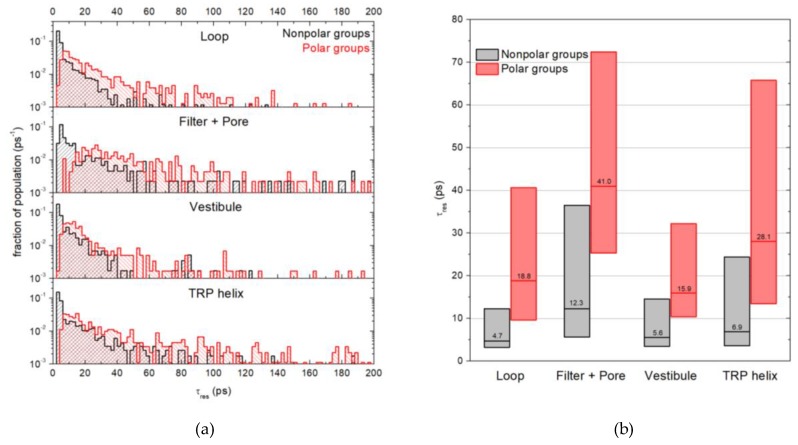
The distributions of the water residence time in hydration shells of polar and nonpolar groups of different TRPV1 domains in a histogram (**a**) and box chart (**b**) representation. Red—polar (and charged) groups, gray—nonpolar groups. Boxes represent interquartile ranges of the corresponding histograms; line and number in each box is the median τ_res_ of the corresponding histogram.

The distributions of water residence time (τ_res_, see Materials and Methods) were obtained separately for polar and nonpolar groups of different protein domains (Figure 5a). Nonpolar groups of the loop, vestibule, and TRP domains showed similar distributions with a narrow peak in the range of short residence times (1–5 ps), which indicates fast water dynamics, and a long tail of larger residence times that corresponds to slowed down dynamics. Polar groups of the same domains do not have the peak of fast dynamics, while the tail decreases slowly compared to that observed for nonpolar groups. Since the pore and filter domains possess a smaller number of hydrated groups, their distributions were merged. In this case, the fast dynamics peak in the distribution for nonpolar groups is less pronounced and extends to 8 ps. In the case of polar groups, the distribution demonstrates slower water dynamics; its left edge is shifted relative to the less confined domains by 5 to 8 ps to the larger residence times’ direction.

In Figure 5b, the same τ_res_ distributions are shown in a box chart representation. The boxes report interquartile ranges of the τ_res_ distributions, with the line and number in each box showing the median τ_res_. For the loop, vestibule, and TRP domains, water molecules show a 3 to 4 times longer residence time near polar groups compared with nonpolar groups (median τ_res_ 16–28 ps and 5–7 ps, respectively). For the pore and filter domains, the similar ratio of τ_res_ occurs (12 and 42 ps, respectively). Furthermore, water residence times in these domains are 1.5 to 3 times longer than in other domains.

**Figure 6:**
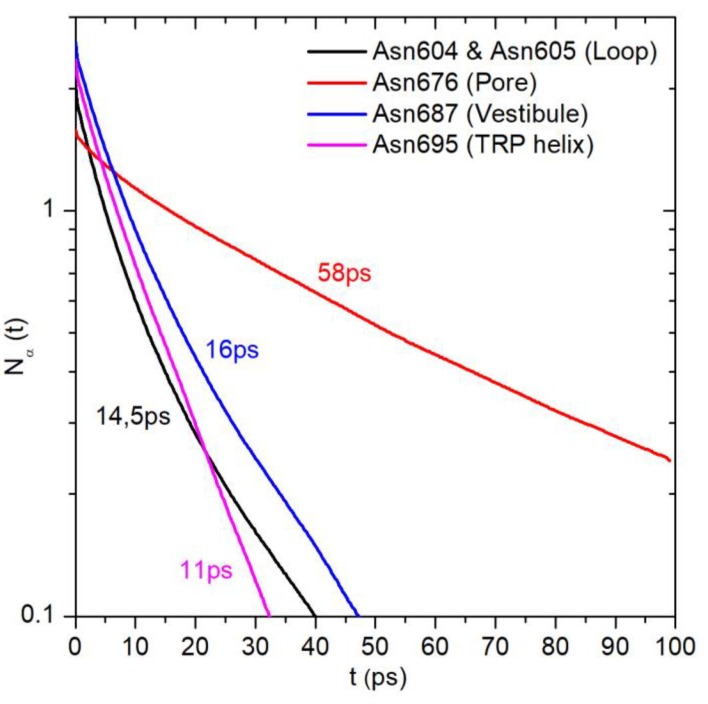
Water survival time correlation functions calculated for the carbonyl oxygen atom of the side chain of asparagine residues (OD1 group) located in different protein regions. The correlation functions were averaged over four identical protein subunits and over four MD trajectories. Corresponding values of τ_res_ are shown near the curves.

Another way to compare the dynamics of confined water in a protein hydration shell is to calculate the water residence time for the same protein group located in different protein regions. The carbonyl oxygen atom of the asparagine residue side chain (OD1 group) was chosen for this purpose. Figure 6 shows water survival time correlation functions and τ_res_ calculated for the OD1 group of the next residues: Asn604 and Asn605 of the loop domain, Asn676 located in the pore, Asn687 in the vestibule, and Asn695 at the TRP helix (see Figure 1). Asn-OD1 groups of the loop, vestibule, and TRP domains reveal similar values of correlation function parameters: Coordination number (N_α_ (*t* = 0), see Materials and Methods) in the range of 2.0 to 2.6 and τ_res_ in the range of 11 to 16 ps. Asn676 located in the confined volume of the pore shows less hydration N_α_(*t* = 0) = 1.6 and slower water dynamics τ_res_ = 58 ps in spite of the same solvent exposure of its polar OD1 group.

**Figure 7:**
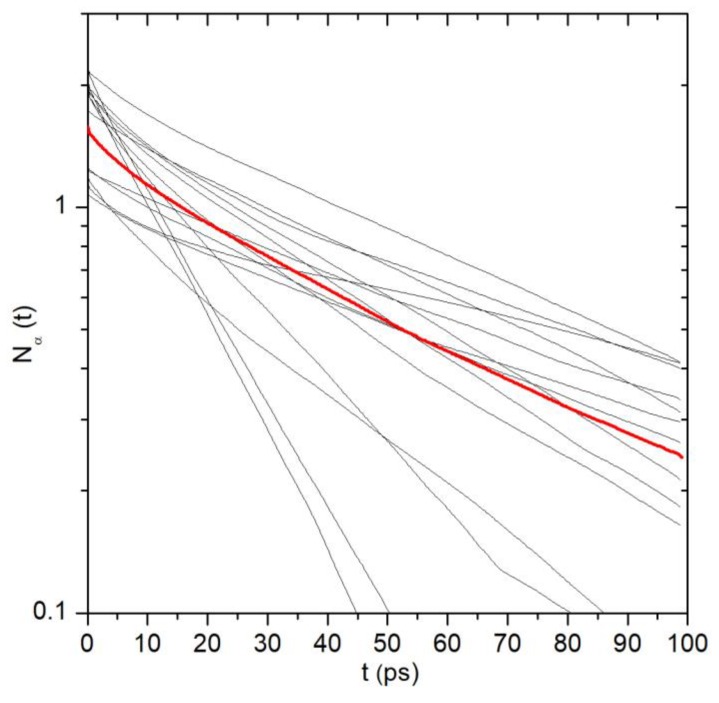
Water survival time correlation functions calculated separately for the Asn676-OD1 group of four protein subunits and four MD trajectories (black curves). Red curve is the average of the black curves.

Averaging over the protein subunits and MD trajectories broadly used in this study is a convenient instrument for the comparison of large protein domains. However, a great variety of properties for the same protein sites or groups should be noted. Figure 7 shows the water survival time correlation functions calculated separately for the Asn676-OD1 group of four protein subunits and four MD trajectories. Despite the fact that they are the same groups of identical subunits, the parameters of water dynamics near the groups vary considerably: τ_res_ from 16 to 128 ps, coordination number from 1.1 to 2.2. Moreover, the atoms of Asn676-OD1 are fully dehydrated in two cases.

The observed variety of the water dynamics is the consequence of two facts that we believe are not the artifacts of MD simulation but represent the natural property of the protein under study. The first one is the asymmetrical structure of TRPV1 that arises from the initial symmetric experimental model due to the flexibility of large protein domains in the course of MD simulations [24]. This is reflected in the asymmetrical spatial distribution of water in the channel (Figure 4). The second one is the flexibility of the side chains of individual residues. These two factors are responsible for heterogeneity of the microenvironment of particular protein groups and affect the water dynamics in their hydration shells.

## 3. Discussion and Conclusions

In this work, we employed MD simulations to study the atomistic resolution dynamics of water molecules confined in the transmembrane ion/water conducting pathway of the temperature sensor protein—TRPV1—channel. The main objective was to explore in detail the behavior of water in a rather unusual nano volume with varying geometry and complex distribution of physico-chemical properties of the internal walls of the pore. Firstly, the solvent-accessible space represents a bottle-like reservoir with two, instead of just one, bottlenecks, linked at the extremities to funnels—vestibules of the channel. The radius of the channel pathway changes from less than one to 4 to 7 Å along the membrane normal (Figure 2). Secondly, the walls of the inner channel are highly hydrophobic in the central part and contain a number of polar protein groups exposed to the pore to varying degrees [24]. Finally, the unique nature of the pore domain is also determined by asymmetrical disposition of the TRPV1 subunits and the highly dynamic character of particular protein polar and nonpolar groups lining the pore. Altogether, this makes the pore in TRPV1 a very interesting object to study the confined water inside—this naturally-occurring system drastically differs from the chemically synthesized artificial porous nano objects, like carbon nanotubes (CNTs), nano slits, and so on.

As expected, the water in the channel showed a noticeable slowdown, which is caused by the limited space (and, therefore, excluded volume effect) and the presence of protein groups capturing waters via hb interactions. Although these effects are commonly observed in any limited nano reservoir, the picture presented here for TRPV1 has some intriguing specific features. Thus, the water “braking” near polar groups of the confined protein domains, like the pore and selectivity filter of TRPV1, leads to an interesting effect of water localization at the particular sites of the protein (Figure 4). These areas (or zones with anomalously high water density) preferably occupy the protein regions, where most of the waters (about 70%) are bound to the protein polar groups. Apparently, such “water blobs” play an important role in the functional activity of TRPV1. In particular, Asn676-OD1 polar groups of four protein subunits may take part in the hydration of the hydrophobic TRPV1 pore by capturing up to six waters near the lower gate of the channel. This, in turn, reduces the free energy barrier for ion and water transport [26].

The effect of water slowdown near the confined protein domains (pore and filter) is evident from the analysis of residence time (τ_res_) distributions obtained for all polar and nonpolar groups (Figure 5). In these domains, the τ_res_ values for water are 1.5 to 3 times larger in comparison with other protein regions. Meanwhile, the influence of water–protein hb interactions on water dynamics can be seen in Figure 5b: The residence time of water near polar groups is 3 to 4 times longer compared to nonpolar groups for all considered protein regions.

It is interesting to note that the τ_res_ distributions for nonpolar groups of the loop, vestibule, and TRP domains look similar to those obtained by Sterpone et al. [27] for water reorientation dynamics within a lysozyme hydration shell. Both sets of distributions reveal a narrow peak that corresponds to relatively fast water dynamics, and a long tail of slow water (Figure 5a). The authors explain the peak by the contribution of water molecules located near hydrophobic and hb donor protein groups, while the tail corresponds to water located near the groups that are confined in pockets and clefts on the protein surface. Most of the latter are hb acceptor groups. Such an assumption agrees with our observation that the tails of the distributions of τ_res_ for polar groups decrease more slowly than for nonpolar groups (Figure 5a). In other words, the slowest water molecules are mainly localized near polar than near nonpolar groups. However, earlier, we showed that waters usually form stronger H-bonds as acceptors than proton donors [28]. So, we suggest, that the slowest water can be a donor or acceptor of hb depending on the type of a particular protein group.

MD simulations also show that the water dynamics in the hydration shell of a particular protein polar group critically depend on the group location (i.e., on its microenvironment). For the polar group OD1 of Asn676 that is confined in the TRPV1 pore, the water τ_res_ value is 3 to 5 times larger than that for the same groups in other Asn residues located in less-confined conditions of the extracellular loop, protein surface (TRP helix), and pore vestibule (Figure 1 and Figure 6). This is caused by thte slowed down dynamics of water molecules near the spatially restrained groups. Since the solvation properties (e.g., expressed in terms of the water-accessible surface, etc.) are very similar for the considered groups, the observed changes in water dynamics can only be explained by the excluded volume effect, which has a much greater impact in confined conditions [27].

The presented dynamic picture of water enclosed in the nano-scale TM pore domain of the ion channel TRPV1 radically differs from those observed earlier in artificial nano objects (see, e.g., [11,13,29] and references therein). Thus, in the latter cases, confined water forms either single-file ordered chains in narrow CNTs and small reverse micelles, various n-gonal ice nanotubes in wider CNTs, or two-dimensional clusters in slit-pore spaces. None of these phenomena were found in the biomolecular system under study. Such unique properties of water in nano spaces formed by membrane proteins (ion channels, receptors, and others) can be explained by the rather flexible walls of the pores and their finely tuned amphiphilic surface “portraits”. In the first case, even a slight instant asymmetry in the packing of protein subunits (including thermal fluctuations of non-covalently bound chains) can lead to serious changes of the geometry of the water “nano pool”. Furthermore, fast conformational dynamics of multiple polar/nonpolar protein groups inside the pore induces a prominent effect on the spatial distribution of the high-density water sites—the example of the residue Asn676 clearly demonstrates this. It should be noted that even such a complex picture is too simplified, since cations (Ca^2+^, Na^+^, K^+^), which are the key players in channel functioning, were not considered here. Their appearance will certainly affect the dynamics of water in the pore domain of TRPV1, but this topic is beyond the scope of the present work.

## 4. Materials and Methods

### 4.1. System and Molecular Dynamics Protocol

The studied system was taken from our previous work [24]. It consisted of the TM-segment of TRPV1 (residues 427–719) in the open state (PDB structure 3J5Q22) embedded into a fully hydrated lipid bilayer with a composition similar to the neuronal membrane: 50% of palmitoyloleoylphosphatidylcholine (POPC), 25% of palmitoyloleoylphosphatidylethanolamine (POPE), and 25% of cholesterol molecules (Figure 1). Chloride ions were added to the system for restoring electroneutrality.

Molecular dynamics (MD) simulations were performed using the GROMACS 2018.5 package [30], Amber99sd-ildn force field [31], and TIP3P water model [32]. Simulations were carried out with an integration time of 2 fs, imposed 3D periodic boundary conditions, and constant temperature (310 K) and pressure (1 bar). For electrostatic interactions, the particle-mesh Ewald summation was used (real space cutoff 10 Å and 1.2 Å grid with fourth-order spline interpolation). A twin-range (10/12 Å) spherical cutoff function was employed to treat van der Waals interactions. Four starting configurations of the system were taken from one of the 500-ns MD trajectories calculated earlier [24]. These configurations were heated to 310 K during the 400-ps MD run with fixed positions of the protein heavy atoms. Then, four unrestrained MD simulations were carried out with a length of 2 ns and the time step between the stored states of 0.25 ps.

### 4.2. MD Data Analysis

Translational dynamics of water molecules along the pore axis (*z*) were characterized by the coefficient, D, that was named the translational dynamics coefficient and calculated as:

(1) $$D\left(z\right)=\frac{1}{6T}\overset{T}{\underset{t=0}{\sum }}\frac{1}{N_{w}\left(z,t\right)}\overset{N_{w}\left(z,t\right)}{\underset{i=0}{\sum }}\Delta R_{i}^{2}\left(z,t\right)$$

where T is the trajectory length (2 ns); N_w_(z,t) is the number of water molecules in a layer from z to z + dz at a moment t (dz = 0.25 Å); ΔR_i_^2^(z,t) is the square displacement over time of 1 ps of the molecules, which are located from z to z + dz at a moment t. Parameter D for bulk water numerically equals its diffusion coefficient (about 0.65 Å^2^/ps for TIP3P water at 310 K) and, at the same time, it shows the local heterogeneity of water dynamics along the pore.

Channel radius along the pore axis (*R*) was calculated as:

(2) R(z)=〈V(z)〉πdz

where <V(z)> is the MD-averaged solvent-accessible volume of a layer from z to z + dz (dz = 0.25 Å). That is, the volume, where the center of a sphere with a radius of 1.4 Å can be placed without overlapping with the protein surface.

To compare the water dynamics near polar and nonpolar groups in the protein domains with various confinement conditions, the residence time of water molecules in the hydration shells of protein groups (τ_res_) was calculated. For this, a survival time correlation function was used [25]:

(3) $$N_{\alpha }\left(t\right)=\frac{1}{T}\overset{N_{w}}{\underset{j=1}{\sum }}\overset{T}{\underset{t^{\prime }=0}{\sum }}p_{\alpha ,j}(t^{\prime },t^{\prime }+t;t_{0})$$

Here, T is the trajectory length (2 ns). N_w_ is the total number of water molecules in the system. p_α,j_(t’, t’ + t; t_0_) is the binary function, which equals 1 if the water molecule, j, continuously stays in the hydration shell of a group, α, during the time interval from t’ to t’ + t or leaves the shell during this interval, but returns for the time no longer than t_0_ = 2 ps. Otherwise, the function equals zero. The radius of hydration shell was taken as 3.5 Å for all protein groups. The value N_α_ (*t* = 0) gives the mean hydration shell occupancy (coordination number) of the group, α.

The calculated N_α_ was fitted by a double exponential function:

(4) $$n\left(t\right)=n_{f}e^{-\;t/\tau_{f}}+n_{s}e^{-\;t/\tau_{s}}$$

where τ_f_ and τ_s_ are the fast and the slow decay constants. Only groups with N_α_ (*t* = 0) ≥ 0.2 were taken for fitting. The latter was performed on the time interval from 0.25 to 100 ps or up to the time when N_α_ reaches the value of 0.1 for rapidly decaying functions. The residence time of water molecules was calculated for each group as:

(5) $$\tau_{res}=\langle t\rangle =\frac{\int_{0}^{\infty }tn\left(t\right)dt}{\int_{0}^{\infty }n\left(t\right)dt}=\frac{n_{f}\tau_{f}^{2}+n_{s}\tau_{s}^{2}}{n_{f}\tau_{f}+n_{s}\tau_{s}}$$

In Figure 2 and Figure 4, origin of the pore axis (z) was taken as the pore center of mass calculated over Cα atoms of residues 642, 643, 644, 645, 671, 675, 676, 679, 680, 683, 686, and 687—those are located in the helices S6 and the selectivity filter. The following geometric criteria were used to define water–protein hb: Donor (D) – acceptor (A) atoms distance ≤ 3.5 Å, the angle D-H-A lies in the range 180° ± 30°.

## Abbreviations

CNT	Carbon nanotube
hb	Hydrogen bond
MD	Molecular dynamics
TM	Transmembrane
TRPV1	Transient Receptor Potential Vanilloid type 1
